# Hydrogen storage in Mg_2_Ni(Fe)H_4_ nano particles synthesized from coarse-grained Mg and nano sized Ni(Fe) precursor

**DOI:** 10.1039/c8ra01963k

**Published:** 2018-05-23

**Authors:** Xi Chen, Jianxin Zou, Shuqing Huang, Guangli He, Ning Zhao, Xiaoqin Zeng, Wenjiang Ding

**Affiliations:** National Engineering Research Center of Light Alloy Net Forming, State Key Laboratory of Metal Matrix Composites, Shanghai Jiao Tong University Shanghai 200240 P. R. China zoujx@sjtu.edu.cn +86-21-34203730 +86-21-54742381; Shanghai Engineering Research Center of Mg Materials and Applications, School of Materials Science and Engineering, Shanghai Jiao Tong University Shanghai 200240 P. R. China; National Institute of Clean-and-Low-Carbon Energy Beijing 102211 P. R. China; Shanghai Light Alloy Net Forming National Engineering Research Center Co.,Ltd. P. R. China

## Abstract

In this work, Mg_2_Ni(Fe)H_4_ was synthesized using precursors of nano Ni(Fe) composite powder prepared through arc plasma method and coarse-grained Mg powder. The microstructure, composition, phase components and the hydrogen storage properties of the Mg–Ni(Fe) composite were carefully investigated. It is observed that the Mg_2_Ni(Fe)H_4_ particles formed from the Mg–Ni(Fe) composite have a diameter of 100–240 nm and a portion of Fe in the Ni(Fe) nano particles transformed into α-Fe nano particles with the diameter of 40–120 nm. DSC measurements showed that the peak desorption temperature of the Mg_2_Ni(Fe)H_4_ was reduced to 501 K and the apparent activation energy for hydrogen desorption of the Mg_2_Ni(Fe)H_4_ was 97.2 kJ mol^−1^ H_2_. The formation enthalpy of Mg_2_Ni(Fe)H_4_ was measured to be −53.1 kJ mol^−1^ H_2_. The improvements in hydrogen sorption kinetics and thermodynamics can be attributed to the catalytic effect from α-Fe nano particles and the destabilization of Mg_2_NiH_4_ caused by the partial substitution of Ni by Fe, respectively.

## Abstract 2

Mg_2_Ni(Fe)H_4_ was synthesized from precursors of coarse grained Mg powder and Ni(Fe) nano particles with improved hydrogen sorption kinetics and thermodynamics as compared to Mg_2_Ni(Fe)H_4_.

## Graphical abstract

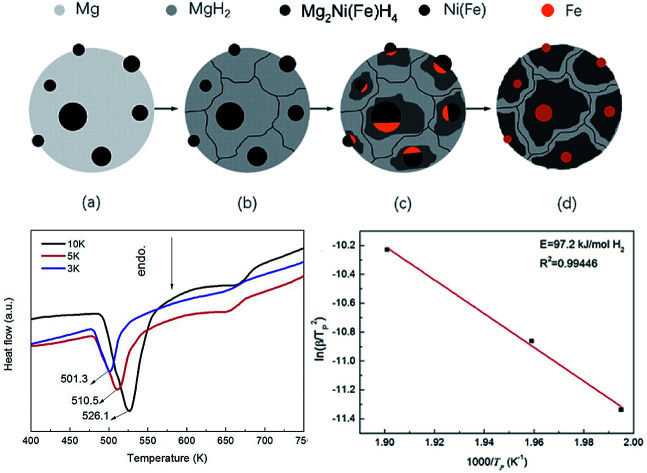

## 1 Introduction

Magnesium hydride has a relatively high hydrogen storage capacity (7.6 wt%), an environmentally friendly nature and low cost, which meets some basic requirements for onboard and stationary applications set by the US DOE. However, the high thermodynamic stability of MgH_2_ (Δ*H* = −75 kJ mol^−1^ H_2_) is a big hurdle for lowering the hydrogen desorption temperature of Mg-based hydrides.^1,2^ It has been established that when Mg alloys contain a non-hydride forming element, the value of hydrogenation enthalpy can be decreased. Therefore, alloying is a traditional and effective strategy for altering the thermodynamics of Mg-based alloys for hydrogen storage.

One of the typical examples is Mg_2_Ni, which can react with H_2_ to form Mg_2_NiH_4_. It should be noted that the formation enthalpy of Mg_2_NiH_4_ (Δ*H* = −64.5 kJ mol^−1^ H_2_) is lower than that of MgH_2_ which allows the former to desorb at lower temperatures than MgH_2_.^3^ Morinaga *et al.* found that hydrogen interacted more strongly with Ni atoms rather than Mg atoms in Mg_2_NiH_4_.^4^ This existence of Ni in Mg_2_NiH_4_ weakened the Mg–H bond as compared to MgH_2_, and leads to a lower formation enthalpy of Mg_2_NiH_4_. The crystallographic and hydrogen storage properties of Mg_2_NiH_4_ were firstly reported by Reilly and Wiswall.^3^ Since single phase Mg_2_Ni cannot be simply obtained by casting as phase separation occurs during solidification,^5,6^ reactive mechanical alloying (RMA), mechanical milling (MM) and high-energy ball milling (HEBM) have been employed to synthesize Mg_2_Ni or Mg_2_NiH_4_.^7–11^ However, it usually takes a long time to prepare Mg_2_Ni or Mg_2_NiH_4_*via* these methods. Therefore, the size of the raw material was reduced to nano-scale to promote the formation of Mg_2_Ni or Mg_2_NiH_4_.^12,13^ Shao *et al.* prepared Mg_2_Ni from Mg and Ni nanoparticles produced by hydrogen plasma-metal reaction.^13^ It was established that at 553 K and 3 MPa hydrogen pressure, Mg_2_NiH_4_ could be generated.

More recently a number of research groups focused on various synthesis methods for doping with additives to further improve the kinetic properties of Mg_2_Ni.^14–17^ Xie *et al.* synthesized nanostructured Mg_2_Ni_1−*x*_Co_*x*_ (*x* = 0.05, 0.1) alloys through hydrogen plasma–metal reaction.^14^ The alloys could absorb 2 wt% H_2_ at 473 K but desorption still required about 573 K. Hara *et al.* synthesized Mg_2_Ni alloys with the addition of Y and depleted Mg by casting and vacuum heating.^15^ The alloys could absorb ∼2–3 wt% H_2_ at 473 K. Lu *et al.* prepared the alloy Mg_90_In_5_Ni_5_ containing the Mg_14_In_3_Ni_3_ phase by sintering followed by mechanical milling.^16^ The alloy has higher reversible hydrogen storage capacity of 3.3 wt% and the minimum dehydrogenation temperature is reduced to 493 K.

Iron is considered as a good catalytic/alloying element to improve kinetics/thermodynamics of Mg-based hydrogen storage materials.^18,19^ In addition, the hydride of Fe is highly unstable, which may be beneficial for the structural reversibility of Mg_2_Ni in dehydriding.^18^ In the present work, the Ni(Fe) nano particles were prepared through an arc plasma method described in previous works.^13,20^ The formation of the Mg_2_Ni(Fe)H_4_ by using Ni(Fe) precursor and its hydrogen sorption kinetic and thermodynamic properties were carefully investigated. Based on the experimental investigations, the mechanisms of the formation and hydrogen sorption behaviors of the Mg_2_Ni(Fe)H_4_ were proposed.

## 2 Experimental

### 2.1 Sample preparation

In this work, the Ni(Fe) composite nano powder was prepared by an arc plasma evaporation apparatus.^20^ The purities of commercially available pure Ni and Fe powders are over 99.5% and the particle size ranges from 50 to 150 μm. The preparation of the powder involves several steps. First, pure Ni and Fe powders were mixed homogeneously with a Ni to Fe weight ratio of 3 : 1. Then the mixture was compressed to cylinders with a diameter of 10 mm and height of 7 mm under a uniaxial pressure of 15 MPa at room temperature. These cylinders were put into the reaction chamber of arc plasma evaporation apparatus filled with mixed gases of 0.01 MPa H_2_ and 0.05 MPa Ar. The Ni(Fe) composite nano powder was produced using arc evaporation with the current set at 150 A. After evaporation and condensation, the powder was passivated in mixed argon and air (7 : 1) for 12 h.

About 2.0 g mixture of the commercially available Mg powder and Ni(Fe) composite nano powder in a 2 : 1 molar ratio was ball milled for 4 h in a planetary ball miller. The Mg powder has a purity of 99.5% and its particle size ranges from 50 to 150 μm. The ball milling was carried out in a 50 ml stainless steel vessel under 0.1 MPa Ar atmosphere at 200 rpm with a ball-to-powder weight ratio of 30 : 1. Then these samples were further heated up to 623 K and 673 K in hydrogen under a pressure of 3.7 MPa. After hydrogenation, samples were cooled down to room temperature and the sample container was evacuated to 10^−3^ Pa.

### 2.2 Characterization

The compositions of the Ni(Fe) and Mg–Ni(Fe) powders were analyzed by using inductive coupled plasma emission spectrometer (ICP). The phase identifications of as-milled and hydrogenated composite powders were carried out by X-ray diffraction (XRD) using a D/max 2550VL/PCX apparatus equipped with a Cu Kα radiation source. The morphology and microstructure of the powders were characterized by a JEM-2100F transmission electron microscope (TEM) equipped with an energy dispersive spectrometer (EDS) micro-analysis and a backscattered electron detector. The hydrogen sorption isotherms at various temperatures were obtained using a Sievert type pressure–composition–temperature (PCT) volumetric apparatus provided by Shanghai institute of microsystem and information technology. The dehydriding behaviors of hydrogenated samples were investigated by using differential scanning calorimetry (DSC, Netzsch STA449F3 Jupiter) under 0.1 MPa Ar atmosphere at heating rates of 3, 5 and 10 K min^−1^ from room temperature to 773 K.

## 3 Results and discussions

### 3.1 Formation of Mg_2_Ni(Fe)H_4_ from Mg and Ni(Fe) powders

**Fig. 1:**
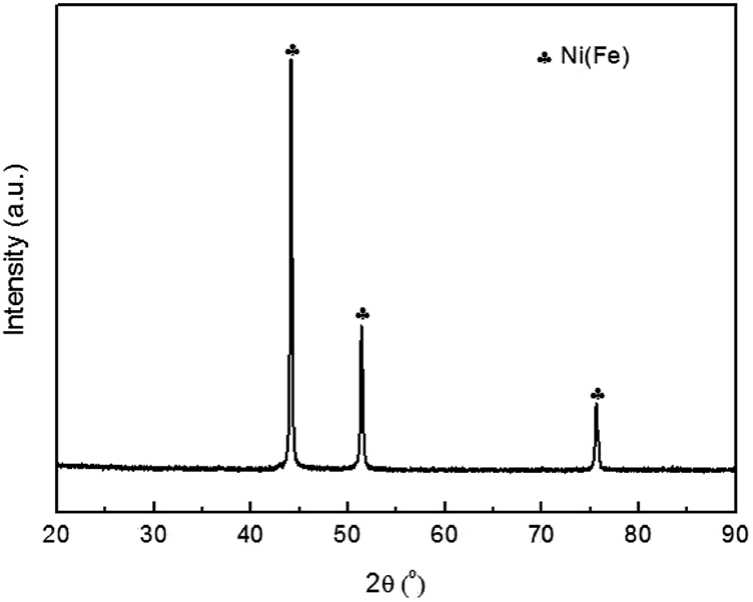
The X-ray diffraction (XRD) pattern of the Ni(Fe) composite powder prepared using arc plasma method.

Fig. 1 shows the XRD pattern of the as prepared Ni(Fe) powder. These peaks correspond to Ni phase but shift to low angles, suggesting the increased lattice parameter. Based on the XRD patterns, the lattice constant of this phase is determined to be: *a* = 0.3548 nm, which is higher than that of Ni (*a* = 0.3535 nm). Such a lattice expansion of Ni can be only attributed to the partial substitution of Ni by Fe. Based on the Scherrer's equation,^21^ the average grain size of Ni(Fe) is determined to be 80 nm.

ICP analysis revealed that Ni content in the as prepared Fe(Ni) composite powder is 61.2 wt%, lower than that in the original mixture (75 wt%). According to the Ohno's model, the vapor generation rate of a metal through hydrogen plasma reaction method is proportional to its reaction parameter (*R*_p_), which can be expressed by the following equation:^22^1
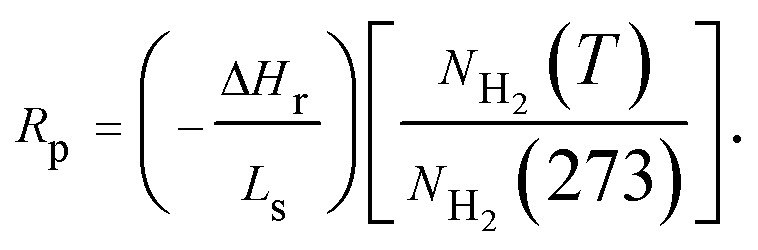
where Δ*H*_r_ is the reaction enthalpy between hydrogen and metal, *L*_s_ is the vaporization heat of the metal at temperature *T*, *N*_H_2__(*T*) and *N*_H_2__(273) refer to the densities of molecular hydrogen in metal at temperature *T* and 273 K, respectively. According to eqn (1), the vapor generation rate mainly depends on the hydrogen affinity to metals (Δ*H*_r_) and vaporization heat (*L*_s_). The reaction enthalpy between H and Fe is quite similar to the one between H and Ni (∼20 kJ mol^−1^ H_2_) while the heat of vaporization for Ni (370.4 kJ mol^−1^) is higher than that for Fe (349.6 kJ mol^−1^). Therefore, the evaporation rate of Ni is lower than that of Fe. This is consistent with the ICP result for which the Ni content in the Ni(Fe) powder is lower than that in the mixed powder before arc evaporation.

**Fig. 2:**
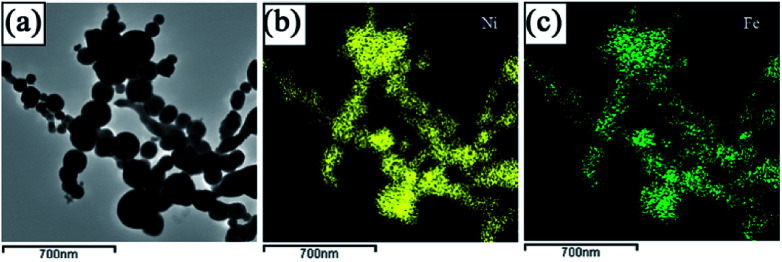
A bright field TEM micrograph of as prepared Ni(Fe) composite powder (a) and the corresponding EDS elemental distribution maps of Ni (b) and Fe (c).

TEM observations have been done on the as prepared Ni(Fe) composite powder. As seen from Fig. 2a, the shapes of the particles in the Ni(Fe) composite powder are mostly spherical and the particle size ranges from 50 nm to 260 nm with an average size of about 80 nm. Such morphology and size distribution were also observed in pure Ni powder produced by arc plasma method.^23,24^ The corresponding elemental distribution maps of Ni and Fe (Fig. 2b and c) confirm the fairly homogeneous distributions of Ni and Fe in the Ni(Fe) powder.

Furthermore, EDS analyses also reveal that the Ni to Fe weight ratio is 59.2 : 40.8, which is in good agreement with the result obtained by the ICP measurement.

**Fig. 3:**
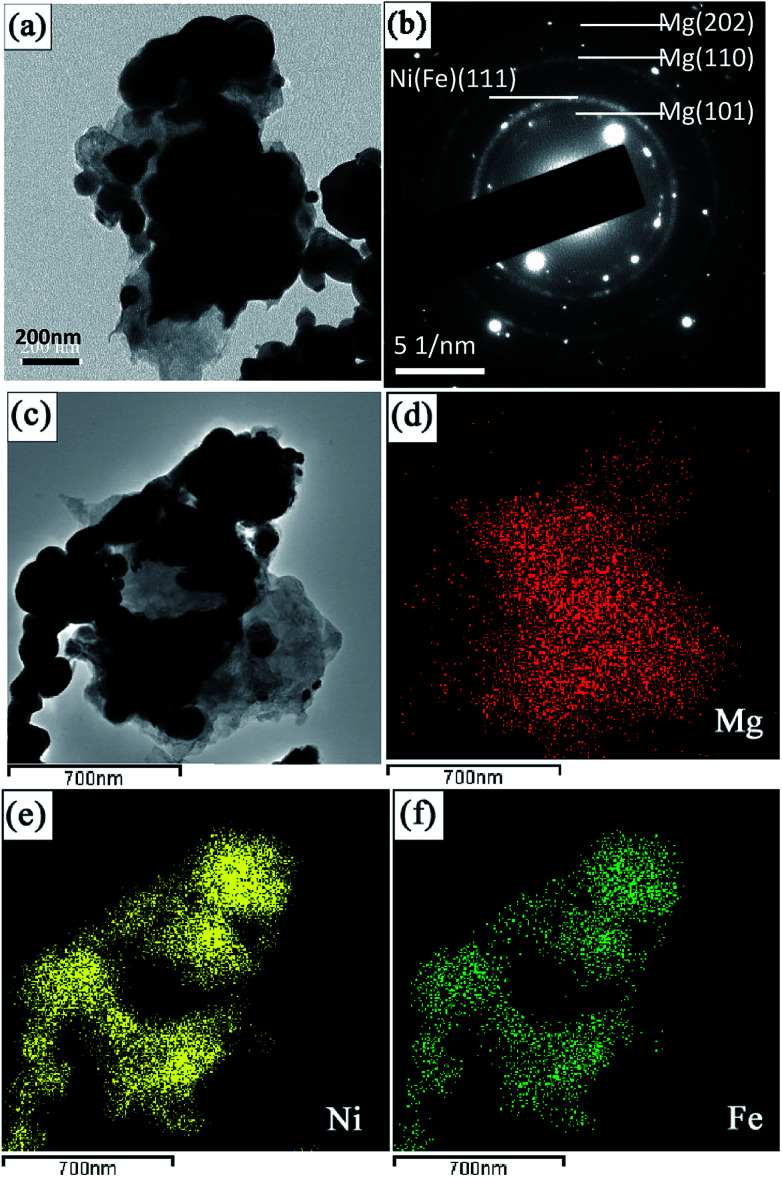
The bright field TEM micrographs of the Mg–Ni(Fe) mixture (a) and (c), the corresponding selected area electron diffraction (SAED) pattern (b), corresponding EDS maps showing the distributions of Mg (d), Ni (e) and Fe (f).

Fig. 3a is the TEM image of the mixture of coarse-grained commercial Mg powder and Ni(Fe) nano powder. It can be seen that there are many smaller particles surrounding those larger ones in this Mg–Ni(Fe) powder. The corresponding SAED pattern (Fig. 3b), with diffraction rings of Mg and Ni(Fe) included, confirms the presence of these phases. The corresponding EDS maps of Mg, Ni and Fe (Fig. 3d–f) confirm the segregations of Ni and Fe elements in the smaller spherical particles and the uniform distribution of Mg element in the bigger irregular ones. Therefore, there are many Ni(Fe) nano particles surrounding coarse-grained commercial Mg particles in the Mg–Ni(Fe) mixture.

**Fig. 4:**
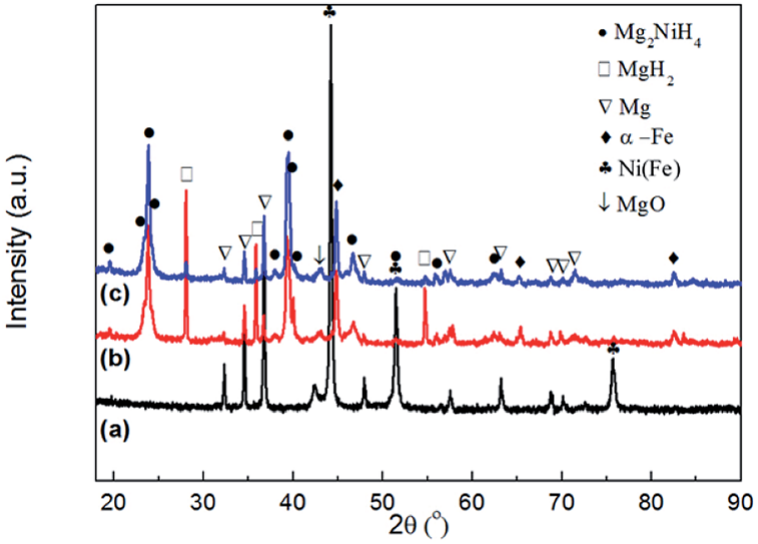
XRD patterns of as milled Mg–Ni(Fe) powder (a), the Mg–Ni(Fe)–H powder hydrogenated at 623 K under 3.7 MPa H_2_ for 48 h (b), the Mg–Ni(Fe)–H powder hydrogenated at 673 K under 3.7 MPa H_2_ for 48 h (c).

Fig. 4 shows the XRD patterns of the Mg–Ni(Fe) samples before and after hydrogenation. From Fig. 4a, the main phases present in the as milled Mg–Ni(Fe) composite powder are Mg and Ni(Fe). The result is consistent with the SAED pattern of the composite powder (Fig. 3b). For the synthesis of Mg_2_NiH_4_, the Mg–Fe(Ni) samples were hydrogenated at 623–673 K under 3.7 MPa H_2_ for 48 h. The phase components of the Mg–Ni(Fe)–H powders under different conditions were examined using XRD and the results were shown in Fig. 4b and c. Based on the RIR analysis of those XRD patterns, the phase components in the hydrogenated powders were calculated and the results were given in Table 1. After hydrogenation at 623 K under 3.7 MPa H_2_ for 48 h, peaks from Mg_2_NiH_4_ and α-Fe appear on the XRD pattern of the Mg–Ni(Fe)–H powder (Fig. 4b). Meanwhile, Ni(Fe) content reduces from 57.8 wt% in as milled Mg–Ni(Fe) powder to 2.0 wt% in the hydrogenated one. Therefore, with the presence of Ni(Fe) in the Mg–Ni(Fe) powder, Mg_2_NiH_4_ is able to be synthesized and then most of Ni(Fe) have been transformed into α-Fe. As the temperature increases from 623 K to 673 K, the diffraction peaks of β-MgH_2_ become weaker and Ni(Fe) could hardly be detected on the XRD pattern of Mg–Fe(Ni)–H powder. Indeed, higher temperature favors the diffusion of H, Mg and Ni atoms, thus, after hydrogenation at 673 K under 3.7 MPa H_2_ for 48 h, the yield of Mg_2_NiH_4_ increases to 61.2 wt% in the Mg–Ni(Fe)–H powder.

**Table 1:** Phase composition (wt%) in as-synthesized samples determined from RIR analysis of XRD data

Sample	Mg–Ni(Fe)	Mg–Ni(Fe)–H (623 K, 3.7 MPa H_2_)	Mg–Ni(Fe)–H (673 K, 3.7 MPa H_2_)
β-MgH_2_	—	27.2	10.2
MgO	—	3.0	3.0
Mg	42.2	10.7	6.8
α-Fe	—	21.3	19.8
Ni(Fe)	57.8	2.0	—
Mg_2_NiH_4_	—	35.8	61.2

**Fig. 5:**
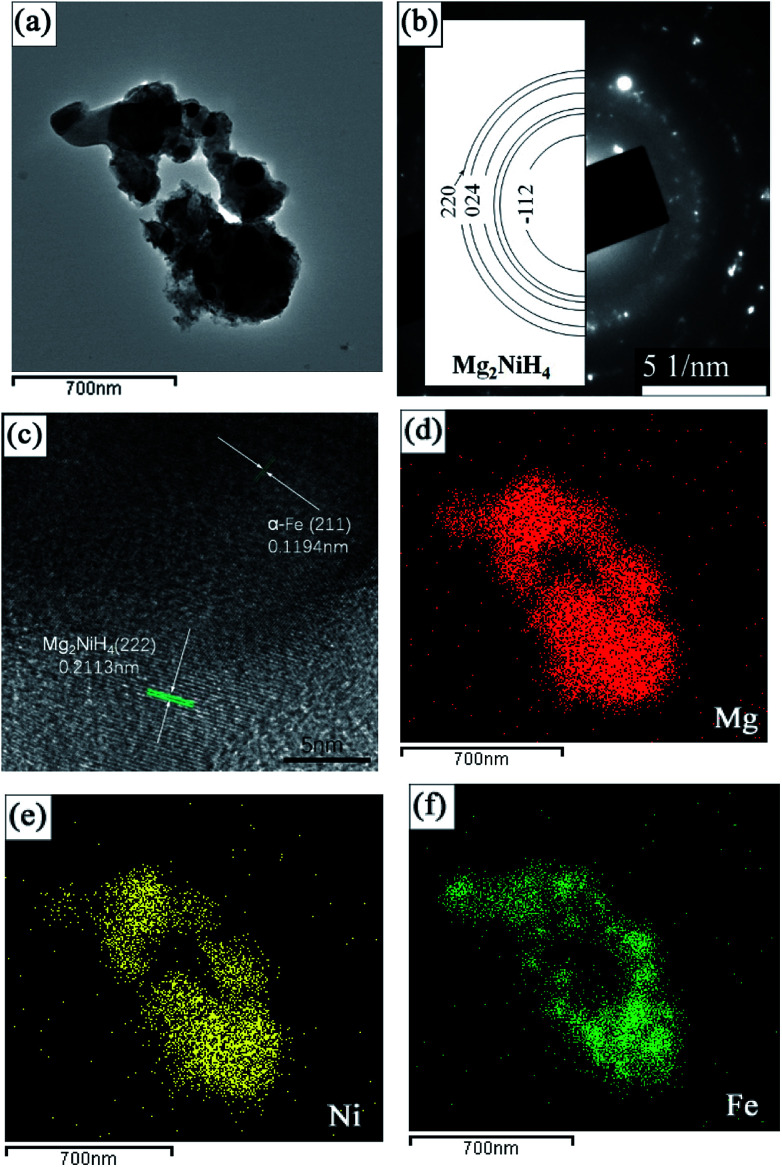
A bright field TEM micrograph of the Mg–Ni(Fe)–H powder prepared at 673 K under a 3.7 MPa hydrogen atmosphere for 48 h (a), the corresponding SAED pattern (b), the corresponding high-resolution TEM (HRTEM) image (c) of the selected zone in (a), and corresponding EDS maps showing the distribution of Mg (d), Ni (e) and Fe (f).

**Fig. 6:**
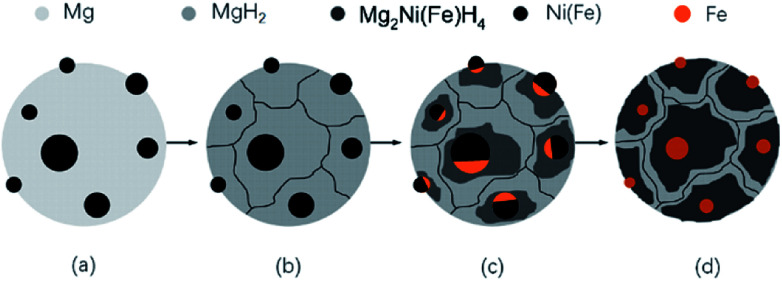
Schematic illustrations showing the formation procedures of the Mg_2_Fe(Ni)H_6_ from coarse-grained Mg powder and γ-Fe(Ni) nano particles.

Fig. 5a presents the typical bright field TEM micrograph of Mg_2_NiH_4_ in the Mg–Ni(Fe)–H powder prepared at 673 K under a 3.7 MPa hydrogen pressure for 48 h. The size of the Mg_2_NiH_4_ nano particles is in the range from 100 to 400 nm. The corresponding SAED pattern is given in Fig. 5b, superimposed with the simulated ring patterns of Mg_2_NiH_4_. The majority of the diffraction pattern can be indexed with Mg_2_NiH_4_ phase. In addition, the HRTEM observation was further carried out to study the microstructure of nano particles in more detail, as shown in Fig. 5c. It is clearly shown that the bigger bright particles are Mg_2_NiH_4_ and the smaller dark particles are α-Fe, according to the measurements of inter-planar spacing. The corresponding EDS maps of Mg, Ni and Fe (Fig. 6d–f) confirm the segregation of Fe element in the smaller spherical particles and the uniform distributions of Ni and Mg elements in the bigger irregular ones. Therefore, after hydrogenation at 673 K under a 3.7 MPa hydrogen pressure for 48 h, Ni atoms diffused from Ni(Fe) nano particles to the coarse-grained Mg particles to form Mg_2_NiH_4_ and then a portion of Fe atoms in the Ni(Fe) nano particles transformed into α-Fe nano particles with smaller particle size. The shapes of the α-Fe nano particles are mostly spherical and the particle size ranges from 40 nm to 120 nm with an average size of about 60 nm. In addition, a portion of Fe atoms have also diffused with Ni atoms to form Mg_2_Ni(Fe)H_4_, as confined by the EDS analyses.

During hydrogenation, two reactions occur in the Mg–Ni(Fe) powder:

2 Mg + H_2_ → MgH_2_,

3 2MgH_2_ + Ni(Fe) → Mg_2_NiH_4_ + α-Fe.

The formation of Mg_2_NiH_4_ requires a long-distance diffusion of not only small H atoms but also the large metallic species Mg and Ni. Owing to the low diffusion rates of Mg and Ni, the formation of the Mg_2_NiH_4_ generally suffers from poor kinetics. In systems based on Mg and Ni that are tailored mainly toward fast sorption kinetics, MgH_2_ would rather form prior to Mg_2_NiH_4_.^13,25^Fig. 6 shows a sketch illustrating the formation of Mg_2_Ni(Fe)H_4_ nano particles. Firstly, the mixed Mg powders in micron scale and Ni(Fe) nano particles are heated in hydrogen, as shown in Fig. 6a. At the beginning of the absorption, because of the catalytic effect from nickel and iron on the dissociation of gaseous hydrogen,^26^ MgH_2_ will form through hydrogen spill over mechanism while Ni(Fe) particles still exists, as seen in Fig. 6b. Then Mg_2_NiH_4_ will nucleate at the phase boundaries between MgH_2_ and Ni(Fe) (Fig. 6c). Due to the partial substitution of Ni by Fe in Ni(Fe) lattice, a portion of Fe atoms have dissolved into the Mg_2_NiH_4_ to form Mg_2_Ni(Fe)H_4_. Consequently, when most of MgH_2_ transformed into Mg_2_Ni(Fe)H_4_ in the Mg–Ni(Fe)–H powder, Fe in the Ni(Fe) particles transformed into α-Fe nano particles with smaller grain sizes surrounding the Mg_2_Ni(Fe)H_4_ particles (see Fig. 6d).

### 3.2 Hydrogen sorption behaviors of the Mg–Ni(Fe) powder

**Fig. 7:**
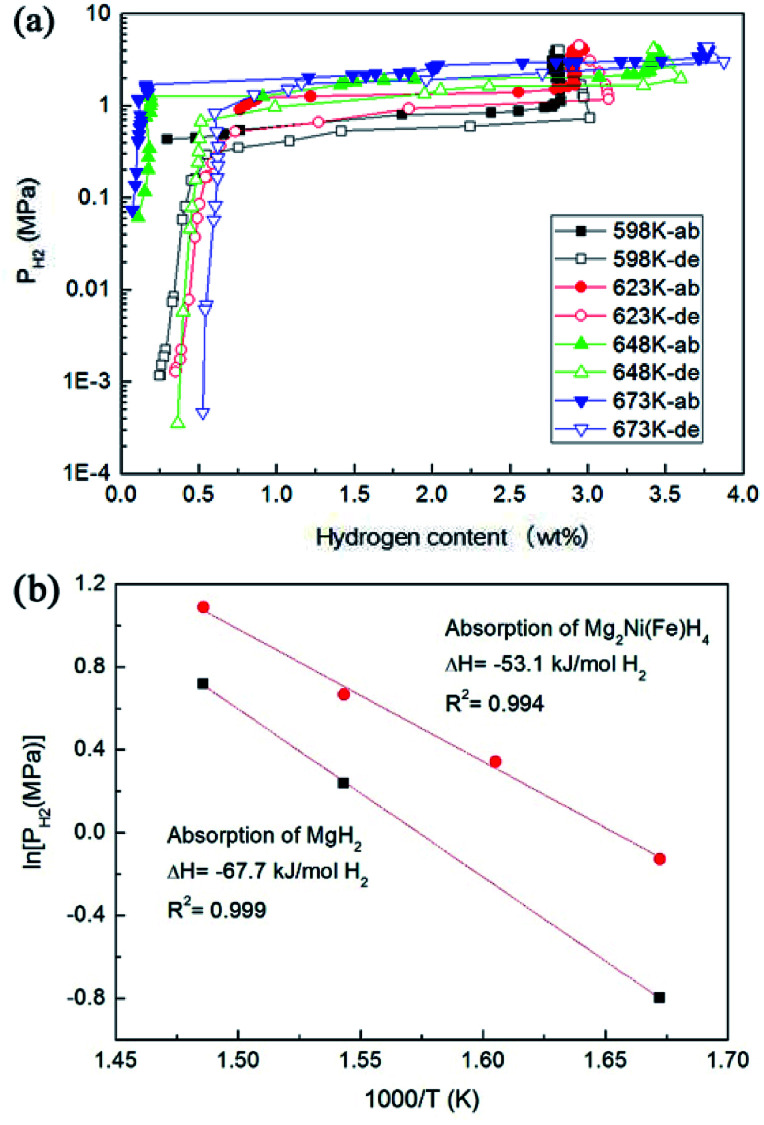
PCT curves at various temperatures of the Mg–Ni(Fe) sample (a), the corresponding van't Hoff plots for MgH_2_ and Mg_2_Ni(Fe)H_4_ in the Mg–Ni(Fe)–H sample (b).

Fig. 7a shows the PCT curves of the hydrogen absorption–desorption processes for the Mg–Ni(Fe) samples at 598, 623, 648 and 673 K. The data obtained from PCT curves are summarized in Table 2. During H_2_ absorption, two plateaus are clearly visible on each profile. According to the XRD results given above, two types of hydrides, MgH_2_ and Mg_2_Ni(Fe)H_4_, formed after hydrogenation. It is worth noting that Mg_2_NiH_4_ has higher equilibrium pressure than MgH_2_ due to its lower thermodynamic stability.^29,30^ The maximum hydrogen absorption capacities at 598, 623, 648 and 673 K are 3.0, 3.1, 3.6 and 3.9 wt%, respectively. It is noticed that the capacities at 598 and 623 K are lower than the theoretical value of 3.6 wt% for Mg_2_Ni. This is due to the existence of MgO, the residual Mg in the hydride Mg–Ni(Fe) composites, as shown in Fig. 4b. However, when the hydrogenated temperature is above 648 K, the capacity is higher than 3.6 wt%. Since the original molar ratio of Mg and Ni(Fe) in the Mg–Ni(Fe) is 2 : 1 and the ICP analysis revealed that Ni content in the Fe(Ni) powder is 61.2 wt%, the molar ratio of Mg to Ni in the Mg–Ni(Fe) is actually 3.3 : 1, higher than 2 : 1. Therefore, after complete hydrogenation, the maximum hydrogen absorption capacities of the Mg–Ni(Fe) can be higher than 3.6 wt%.

**Table 2:** Data obtained from pressure–composition isotherms of the Mg–Ni(Fe) sample at various temperatures

Temperature (K)	Low plateau of absorption (MPa)	High plateau of absorption (MPa)	Maximum hydrogen content (wt%)
598	0.45	0.88	3.0
623	—	1.41	3.1
648	1.27	1.95	3.6
673	2.05	2.97	3.9

**Fig. 8:**
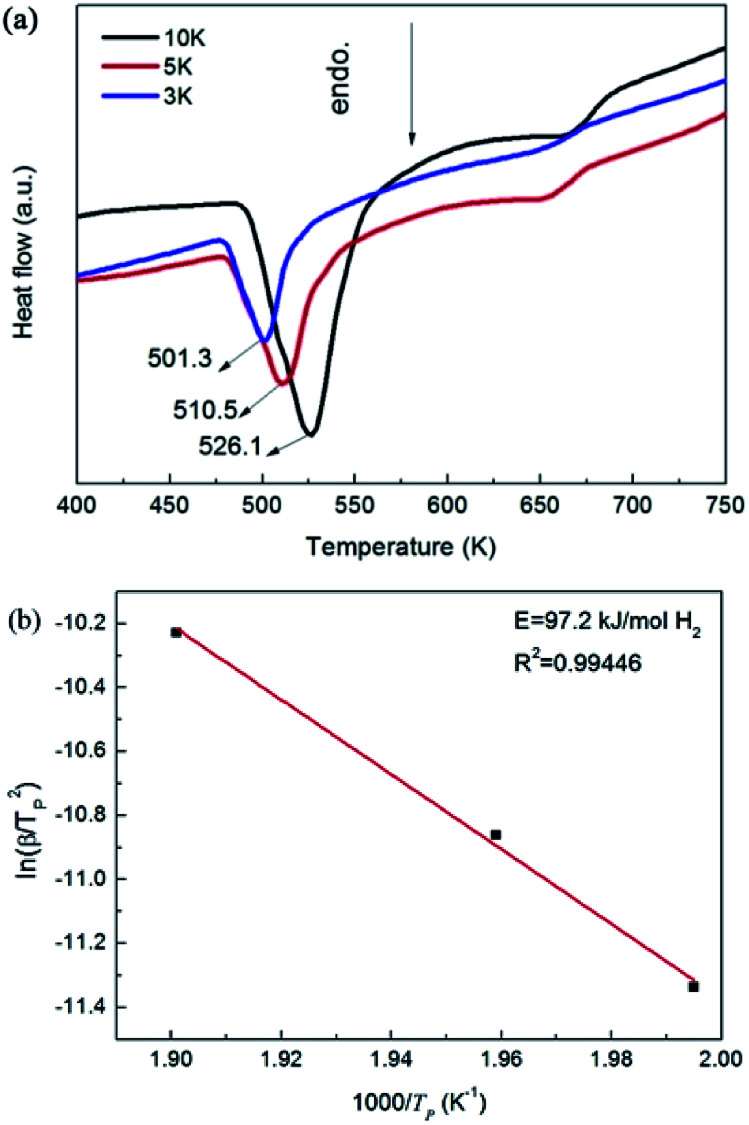
DSC profiles of the Mg–Ni(Fe)–H sample measured at different heating rates (a) and the corresponding ln(*β*/*T*_p_^2^)–1000/*T*_p_ plot (b).

The van't Hoff plot (ln *P versus* 1/*T*) is used to estimate the thermal stability of hydrides, as shown in Fig. 7b. Table 3 presents the value of the formation enthalpy (Δ*H*) for the Mg_2_Ni(Fe)H_4_ in this work and the ones for the Mg_2_NiH_4_ reported in literature. According to the linear fittings of ln *P versus* 1000/*T* in Fig. 8b, the van't Hoff equations for the hydrogenation are ln(*P*_low_) = −6.392 × 10^3^/*T* + 10.57 for MgH_2_ and ln(*P*_high_) = −8.138 × 10^3^/*T* + 12.81 for Mg_2_Ni(Fe)H_4_. The hydrogenation enthalpies (Δ*H*_ab_) for MgH_2_ and Mg_2_Ni(Fe)H_4_ are therefore calculated to be −67.7 and −53.1 kJ mol^−1^ H_2_, respectively. In contrast, Pourabdoli *et al.* reported that the integral heat of H_2_ desorption for the MgH_2_-10 wt% (9Ni–2Mg–Y) nano-composite was about 78 kJ mol^−1^ H_2_ measured by using adsorption micro-calorimetry.^31^ Therefore, the formation enthalpy of Mg_2_Ni(Fe)H_4_ is obviously higher than those reported in literature. Van Setten *et al.* have studied the influence of transition metals (Cu, Fe and Co) on the structural and hydrogen sorption properties of Mg_2_NiH_4_ using first-principle based calculations.^18^ Doping with Co or Cu leads to octahedron is distorted. The hydrogen tetrahedra around such Ni atoms are distorted with Ni–H distances from 1.51 to 1.80 Å, whereas in undoped Mg_2_NiH_4_ they are between 1.56 and 1.58 Å. Therefore, the highest hydriding enthalpy, −55.5 kJ mol^−1^ H_2_, is found for the Fe doped Mg_2_NiH_4_.^18^ This value is higher than the simulated value for Mg_2_NiH_4_ (−63.5 kJ mol^−1^ H_2_). Therefore, the results present in this work also confirm that doping with Fe would remarkably destabilize Mg_2_NiH_4_ through increasing the formation enthalpy. In addition, the thermodynamics of marginally stable compounds, whereas doping with Fe leads to an unstable compound. In the Fe doped Mg_2_NiH_4_, the hydrogenation of MgH_2_ in the Mg–Ni(Fe) is also slightly improved by the addition of Ni(Fe) nanoparticles, which is in accordance with recent experimental results and theoretical calculations. For instance, the hydriding enthalpies of Mg–Ni nanocomposite coprecipitated from solution and MgH_2_–Ni/Ti prepared by ball milling were reduced to −70.0 and −67.8 kJ mol^−1^ H_2_.^32,33^ Dai and Shevlin carried out theoretical calculations and found that the significant electronic density donation from the H^−^ ions to an empty d-state of the Ni dopant was able to reduce the H^−^ anion charge to weaken the hydrogen bonding.^34,35^ Thus, doping with Ni would also remarkably destabilize MgH_2_ to improve the thermodynamics of MgH_2_.

**Table 3:** The formation enthalpy value of Mg_2_Ni(Fe)H_4_ in the Mg–Ni(Fe)–H sample and the ones of Mg_2_NiH_4_ reported in the literatures^25,27–29^

Formation (Δ*H*) (kJ mol^−1^ H_2_)	Reference
−53.1	This work
−67.5	25
−64.6	27
−66.3	28
−63.2	29

Fig. 8a shows DSC curves obtained by heating the Mg–Ni(Fe)–H powder up to 723 K at heating rates of 3, 5 and 10 K min^−1^ under Ar. These curves suggest a two-step desorption behavior by showing two endothermic peaks. The first one is a strong and broad endothermic peak in the lower temperature range, while the second one is a relatively weaker peak in the higher temperature range. Clearly, the strong peak corresponds well to the decomposition of Mg_2_Ni(Fe)H_4_ while the weak one results from the decomposition of the MgH_2_. The peak dehydrogenation temperatures of the Mg_2_Ni(Fe)H_4_ in the Mg–Ni(Fe)–H powder at heating rates of 3, 5 and 10 K min^−1^ are 501.3, 510.6 and 526.1 K, respectively. Zou *et al.* prepared the Mg-rich Mg–Ni ultrafine particles through the arc plasma method.^27^ DSC analyses showed that there are three endothermic peaks appeared at 513, 643 and 668 K, which correspond to the phase transformation of Mg_2_NiH_4_ from its low temperature form to the high temperature form, the dehydriding of Mg_2_NiH_4_ and MgH_2_, respectively. Therefore, the peak dehydrogenation temperature of the Mg_2_Ni(Fe)H_4_ in the Mg–Fe(Ni)–H powder is 117 K lower than that of the Mg_2_NiH_4_ in the Mg-rich Mg–Ni ultrafine particles. In addition, it is noteworthy that the peak dehydrogenation temperatures of the Mg_2_Ni(Fe)H_4_ in the Mg–Ni(Fe)–H powder is indeed in the temperature range of the phase transformation of Mg_2_NiH_4_.^36,37^ Thus, doping with Fe would improve the hydrogen desorption properties of Mg_2_NiH_4_ through promoting the decomposition of the low temperature form of Mg_2_NiH_4_.

To further study the desorption behavior of the Mg–Ni(Fe)–H powder, the dehydrogenation activation energy (*E*_de_) was calculated by using Kissinger's method:^16,20^4
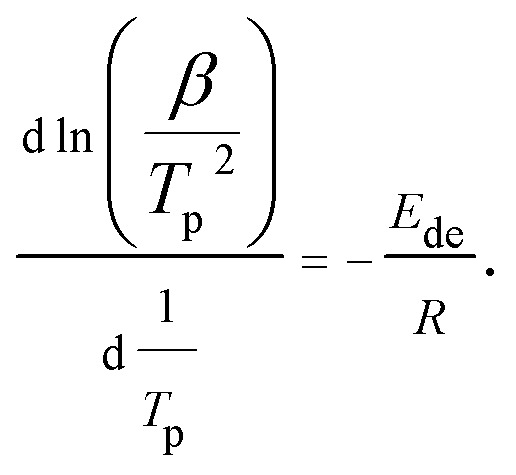
where *β* is the heating rate, *T*_p_ is the peak temperature, and *R* is the gas constant. Just as expected, the peaks of dehydrogenation shift toward higher temperature as the heating rate increases. According to eqn (4), the *E*_de_ value of the Mg_2_Ni(Fe)H_4_ in the Mg–Ni(Fe)–H is estimated to be 97.2 kJ mol^−1^ H_2_. It is obviously lower than those reported by Hur and Cermak,^38,39^ which are 109 and 113 kJ mol^−1^ H_2_, respectively. Giusepponi *et al.* found that the higher coordination of iron compared to magnesium in the hydride was able to destabilize the crystalline structure and raise the probability of the H diffusion toward the interface.^40^ Recent research works have clearly shown that Fe nanoparticles play the role of catalyst for the hydrogen absorption–desorption in Mg. Polanski *et al.* have reported that the 2Mg + Fe nanocomposite can absorb 1.1 wt% hydrogen at 303 K due to the catalytic effect of nano Fe particles on the hydrogen absorption in Mg.^41^ Bassetti *et al.* have prepared MgH_2_–Fe nanocomposites, which can release about 5 wt% hydrogen in about 600 s at 573 K due also to the catalytic effect from Fe.^42^ Therefore, a portion of Fe in the Ni(Fe) nano particles transformed into α-Fe nano particles in the Mg–Ni(Fe)–H, which also improved the dehydrogenation kinetics of Mg_2_NiH_4_.

## 4 Conclusions

In the present work, the formation of Mg_2_Ni(Fe)H_4_ in the mixed precursors of the coarse-grained Mg powder and the Ni(Fe) nano powder prepared through arc plasma method was investigated. The microstructure, composition, phase components and hydrogen storage properties of the Mg–Ni(Fe) powder were characterized and the main results are as follows.

(1) After sintering of the Mg–Ni(Fe) powder at 673 K under 3.7 MPa H_2_ for 48 h, the yield of Mg_2_Ni(Fe)H_4_ reached 61.2 wt% and a portion of Fe atoms in the Ni(Fe) nano particles transformed into α-Fe nano particles.

(2) Mg_2_Ni(Fe)H_4_ shows lower hydrogen desorption temperature and better desorption kinetics when compared to the Mg_2_NiH_4_ obtained from arc plasma evaporated Mg–Ni powder. The peak dehydrogenation temperature of Mg_2_Ni(Fe)H_4_ is reduced to 501.3 K and the dehydrogenation activation energy is reduced to 97.2 kJ mol^−1^ H_2_.

(3) The formation enthalpy of Mg_2_Ni(Fe)H_4_ is determined to be −53.1 kJ mol^−1^ H_2_, higher than the values reported in literature for Mg_2_NiH_4_. The improvements in hydrogen sorption kinetics and thermodynamics can be attributed to the catalytic effect of α-Fe nano particles surrounding the Mg_2_Ni(Fe)H_4_ and the destabilization effect caused by Fe substitution for Ni in the Mg_2_Ni(Fe)H_4_, respectively.

## Conflicts of interest

There are no conflicts to declare.

## Supplementary Material

## References

[cit1] Cheng F., Tao Z., Liang J., Chen J. (2012). Chem. Commun..

[cit2] Shao H., Xin G., Zheng J., Li X. (2012). Nano Energy.

[cit3] Reilly J. J., Wiswall R. H. (1968). Inorg. Chem..

[cit4] Morinaga M., Yukawa H. (2002). Mater. Sci. Eng., A.

[cit5] Massalski T. B. , Binary Alloy Phase Diagrams, American Society for Metals, 1986

[cit6] Nayeb-Hashemi A. A., Clark J. B. (1985). J. Phase Equilib..

[cit7] Koch C. C. (1997). Nanostruct. Mater..

[cit8] Huot J., Enoki H., Akiba E. (2008). J. Alloys Compd..

[cit9] Singh A. K., Srivastava O. N. (1995). J. Alloys Compd..

[cit10] Zaluski L., Zaluska A., Ström-Olsen J. O. (1995). J. Alloys Compd..

[cit11] Varin R. A., Czujko T. (2002). Mater. Manuf. Processes.

[cit12] Hampton M. D., Lomness J. K., Giannuzzi L. A. (2002). Int. J. Hydrogen Energy.

[cit13] Shao H., Liu T., Li X., Zhang L. (2003). Scr. Mater..

[cit14] Xie L., Shao H., Wang Y., Li Y., Li X. (2007). Int. J. Hydrogen Energy.

[cit15] Hara M., Morozumi S., Watanabe K. (2006). J. Alloys Compd..

[cit16] Lu Y., Wang H., Liu J., Ouyang L. Z., Zhu L., Zhang D., Zhu M. (2015). J. Phys. Chem. C.

[cit17] Ouyang L. Z., Cao Z. J., Wang H., Liu J. W., Sun D. L., Zhang Q. A., Zhu M. (2013). Int. J. Hydrogen Energy.

[cit18] VanSetten M. J., Wijs G. A. (2007). Phys. Rev. B: Condens. Matter Mater. Phys..

[cit19] Shevlin S. A., Guo Z. X. (2013). J. Phys. Chem. C.

[cit20] Zou J. X., Zeng X. Q., Ying Y. J., Chen X., Guo H., Zhou S., Ding W. J. (2013). Int. J. Hydrogen Energy.

[cit21] Patterson A. L. , X-ray and Neutron Diffraction, 1966, p. 243

[cit22] Ohno S., Uda M. (1984). J. Jpn. Inst. Met..

[cit23] Cui Z. L., Dong L. F., Hao C. C. (2013). Adv. Mater. Res..

[cit24] Cui Z., Zhang Z., Hao C., Dong L., Meng Z., Yu L. (1998). Thin Solid Films.

[cit25] Shao H. Y., Xu H., Wang Y., Li X. G. (2004). Nanotechnology.

[cit26] Liang G., Huot J., Boily S., VanNeste A., Schukz R. (1999). J. Alloys Compd..

[cit27] Zou J. X., Sun H. Q., Zeng X. Q., Ji G., Ding W. J. (2012). J. Nanomater..

[cit28] Rönnebro E., Jensen J., Noréus D., Bjerrum N. (1999). J. Alloys Compd..

[cit29] Nomura K., Akiba E., Ono S. (1981). Int. J. Hydrogen Energy.

[cit30] Liang G., Boily S., Huot J., Neste A., Schulz R. (1998). J. Alloys Compd..

[cit31] Pourabdoli M., Raygan S., Abdizadeh H., Uner D. (2013). Int. J. Hydrogen Energy.

[cit32] Liu Y. N., Zou J. X., Zeng X. Q., Wu X. M., Li D. J., Ding W. J. (2014). J. Phys. Chem. C.

[cit33] Lu H. B., Poh C. K., Zhang L. C., Guo Z. P., Yu X. B., Liu H. K. (2009). J. Alloys Compd..

[cit34] Dai J. H., Song Y., Yang R. (2010). J. Phys. Chem. C.

[cit35] Shevlin S. A., Guo Z. X. (2013). J. Phys. Chem. C.

[cit36] Post M. L., Murray J. J. (1987). J. Less-Common Met..

[cit37] Li L. Q., Akiyama T., Yagi J. (1999). Intermetallics.

[cit38] Hur T. H., Han J. S., Kim J. H., Kim B. K. (2011). J. Nanosci. Nanotechnol..

[cit39] Cermak J., Kral L. (2009). Defect Diffus. Forum.

[cit40] Giusepponi S., Celino M. (2013). Int. J. Hydrogen Energy.

[cit41] Polanski M., Bystrzycki J., Varin R. A., Plocinski T. (2001). Int. J. Hydrogen Energy.

[cit42] Bassetti A., Bonetti E., Pasquini L., Montone A., Grbovic J., Vittori Antisari M. (2005). Eur. Phys. J. B.

